# A Review Study of a Green Diet and Healthy Ageing

**DOI:** 10.3390/ijerph18158024

**Published:** 2021-07-29

**Authors:** Ben Y. F. Fong, Wang-Kin Chiu, Wendy F. M. Chan, Ting Yu Lam

**Affiliations:** 1 Division of Science, Engineering and Health Studies, College of Professional and Continuing Education, The Hong Kong Polytechnic University, Hong Kong, China; ben.fong@cpce-polyu.edu.hk (B.Y.F.F.); wendy.fm.chan@cpce-polyu.edu.hk (W.F.M.C.); 2 Centre for Ageing and Healthcare Management Research, School of Professional Education and Executive Development, The Hong Kong Polytechnic University, Hong Kong, China; tingyuuullll@gmail.com

**Keywords:** green diet, Mediterranean diet, vegetarian diet, dietary pattern, healthy ageing, chronic conditions, salt intake

## Abstract

Nowadays people are living longer, and there has been a substantial growth in the global elderly population in the past decades. While life expectancy is increasing, there are growing concerns towards the heavy financial and social burdens related to chronic diseases among the elderly. These have been critical health care issues, and healthy ageing is considered a top priority in public health. Diet and eating habits are crucial factors contributing to healthy ageing. These important aspects have attracted much attention in health research, particularly in consideration of the causes and management of chronic conditions which affect most elder adults in the world. Recently, a growing number of investigations have reported significant findings on the association of reduction in the risks of chronic non-communicable diseases with plant-based diets. Meanwhile, there have been worldwide initiatives and programmes implemented for reduction of salt intake. A green diet, which emphasises the consumption of a diet rich in plant foods with minimal portions of red or processed meat and reduced salt intake, is advocated with due consideration to the importance of sustainable environment and healthy ageing. This paper highlights a brief review of the recent advance of knowledge in diet and health, its effects on the elderly and the significance of a green diet on healthy ageing. Implications for a green diet and recommendations for future research are also discussed.

## 1. Introduction

With the rise in global life expectancy and a decline in fertility, population ageing has become an international phenomenon. Over 700 million people in the world were above the age of 65 in 2020 [1]. The number of elderly will continue to grow, with it expected to reach over 1.5 billion by 2050. This means that one in six people worldwide will be aged 65 years or above [1]. Hong Kong, as the region having the highest life expectancy [2], is no exception. It is projected that the proportion of elderly in Hong Kong will increase from about 18% in 2019 to 38% in 2069 [3].

Ageing is the major attributing factor for most common chronic diseases [4]. Hajat and Stein (2018) have found the presence of one or more chronic diseases in about 70% of the elderly in developed countries [5]. Notably, with concurrent gains in life expectancy, there is a worldwide substantial shift from communicable diseases to chronic non-communicable diseases driven by the growing ageing population [6]. Taking Hong Kong as a reference example of the region having the highest life expectancy [2], over half of people with one or more chronic diseases are aged 65 years or above [7]. The common chronic diseases are hypertension, diabetes and heart diseases. Prevalence of these diseases is 9.9%, 4.4% and 2% of the total population, respectively [7]. 60% of people worldwide die from major non-communicable diseases, including cardiovascular diseases (CVD), cancers, chronic lung diseases and diabetes [5].

With the ever-growing ageing population worldwide, the heavy financial and social burdens related to chronic diseases are major concerns, and healthy ageing is considered a public health priority [8]. Over the years, emerging studies with promising findings have reported the health benefits of a reduction in the risk of chronic non-communicable diseases associated with plant-based diets [9,10]. Meanwhile, there have been worldwide initiatives for consuming less salt [11,12]. Although sodium is an essential nutrient, excessive daily ingestion of sodium can lead to hazardous health effects by increasing the risk of high blood pressure, CVD, stroke and renal disease [13,14,15], all of which are of high prevalence among the elderly. In addition to the choice of appropriate food sources, healthy dietary practice is essential to prevent excessive daily salt intake. Salt in the diet can come from various sources including those added to the food during cooking and processing, or added by individuals at the dining table. Even a person living primarily on diets rich in plant foods can have daily salt intake well exceeding the suggested limit for good health. The situation worsens when it comes to the elderly due to degenerated sensitivity to saltiness [16]. Therefore, it is considered that the adoption of diets rich in plant foods and the reduction of salt intake are two very important components of a healthy diet for the elderly. 

A green diet means the consumption of a diet rich in plant foods with minimal portions of meat [17], while a vegetarian diet emphasises dietary patterns based on the consumption of plant-based food without animal meat [18]. Meanwhile, a green diet also focuses on the importance of healthy dietary practices such as reduced salt intake during the consumption of plant-based food. It is also worth noting that a green diet emphasises the benefits of food origins to be environmentally responsible [17]. The word “green” also implies a consumption of food in an appropriate amount to prevent food wastage. The advocation of a green diet is not just focused on the consumption of plant-based food, but at the same time addresses the significance of a sustainable green environment, which is fundamental to healthy ageing [19]. In this paper, we will give a brief review to highlight the recent advance of knowledge in these two major aspects. This perspective starts with a presentation of a general overview of food categories and the health impacts of dietary habits, which is followed by a short review summarising recent significant findings. Further insights on healthy ageing as well as recommendations and implications for a green diet and the reduction of salt intake will also be discussed.

## 2. Materials and Methods

### 2.1. Literature Search

This brief systematic review was conducted with reference to the Preferred Reporting Items for Systematic Reviews and Meta-analyses (PRISMA) guidelines [20]. Searches were performed in two electronic databases: Medline and CINAHL. The search strategies employed the use of terms including green diet, Mediterranean diet, vegetarian diet, dietary pattern, elderly, ageing (aging) and older adults. The search strings were used in combinations in each electronic database and retrieved articles were further extracted to EndNote (version 20) (Clarivate, Philadelphia, PA, USA). 

### 2.2. Study Selection

Restrictions on publication year and language have been applied. The time interval of publication years was from January 2011 to December 2020 and any non-English studies were excluded. If a full-text version could not be obtained, the paper was also excluded. The theoretical framework of the present review is based on two important factors related to healthy ageing, which are the prevalence of chronic diseases and the ageing decline of cognitive functions [21]. The surging ageing population has further intensified the prominent need to address the dietary patterns related to healthy ageing. Considering the prevalence of multimorbidity and cognitive impairment among the elderly, recent findings from the study on dietary patterns and the elderly will form the basis for future investigations related to healthy ageing. In this systematic search, relevant findings will be reviewed and the dietary patterns of healthy ageing will be further discussed. The eligibility criteria for the review included controlled trials and longitudinal studies which investigated the effect of adherence to a plant-based diet on the health of the elderly, with emphasis on major chronic diseases and cognitive impairment. The age group and sample size of the study were also considered. For the current review, the exclusion criteria were: (1) studies not including the elderly group as the participants; (2) studies with limited sample size; and (3) studies not addressing healthy ageing. 

## 3. Results

### Search Results

**Figure 1:**
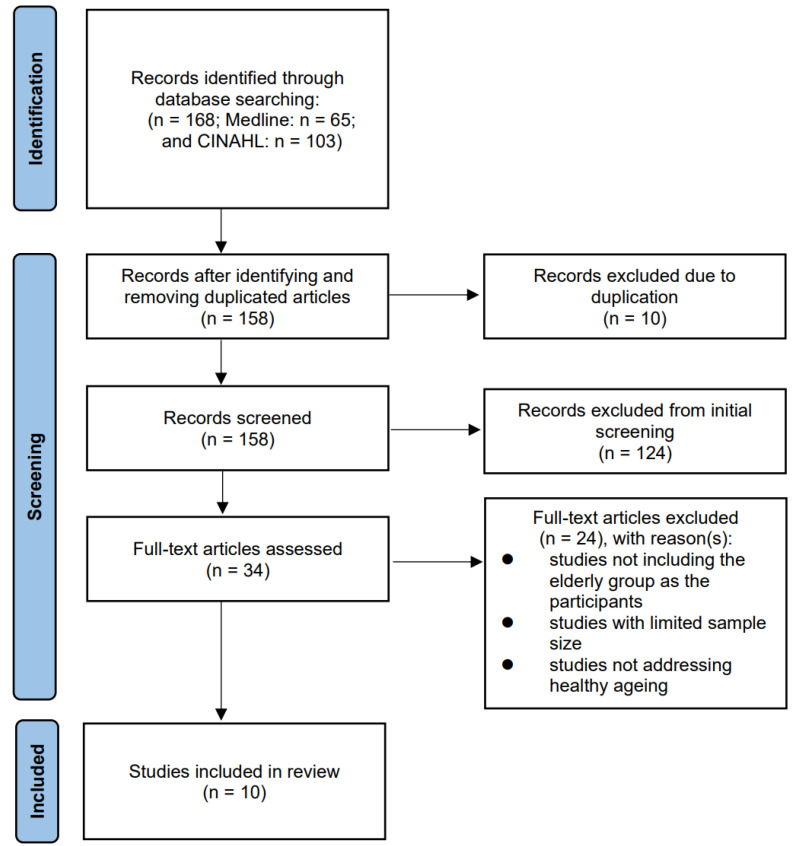
PRISMA flow diagram highlighting the study selection process.

The selection process of relevant studies for this paper is summarised in Figure 1. The search strategy described in Section 2 identified 168 articles through the corresponding searches in two database systems. 10 duplicates were removed. After initial screening and further content review, 148 articles were removed considering the sample size, age group of participants and types of interventions. Consequently, 10 articles were included in this review [22,23,24,25,26,27,28,29,30,31]. The summary table of the 10 studies is given in Table 1.

## 4. Overview and Discussion

### 4.1. Overview of Food Categories in Daily Life

Food is essential to sustain life but may affect body conditions, including physical health. All kinds of food provide energy and nutrients that can maintain body functions and repair body tissues. Nutrients are classified into two groups: macronutrients and micronutrients.

Macronutrients include carbohydrates, protein, dietary fats and fibre. Carbohydrates are mainly divided into three types: monosaccharides, disaccharides and complex carbohydrates. They are the main source of energy in daily diets. Proteins are composed of different amino acids and essential amino acids can only be taken from food. They are mainly used in the growth and repair of body tissues [32]. 

Dietary fat is one of the sources of calories. It can be divided into monounsaturated fats, polyunsaturated fats, saturated fats and trans fats. Unsaturated fats can reduce the level of low-density lipoprotein (“bad” cholesterol) and, thereby, preventing atherosclerosis, arterial blockage, while saturated fats and trans fats can increase “bad” cholesterol levels, leading to the blockage of arteries. Dietary fat enables the body to maintain heat to keep it warm at extreme temperatures and protects organs from shocks [32].

Dietary fibre is the part found in plants that cannot be digested by the body. Generally, it can be divided into two types: soluble fibre and insoluble fibre. Water-soluble fibre can lower blood cholesterol levels and stabilise blood sugar levels, while insoluble fibre can improve bowel function and prevent constipation [33].

Micronutrients include vitamins and minerals. Vitamins are divided into two types: water-soluble and fat-soluble. Different types of food can provide different vitamins for different body functions, such as preserving healthy skin and hair, and building bones. Minerals can regulate many body functions such as fluid balance, muscle contraction and nerve signal transmissions [33].

Whole food and processed food are two distinct food categories in daily life. Whole food refers to natural food that has not been processed, and without any artificial substance added to it [34]. It includes grains, vegetables, fruits, meat, fish, egg, legumes, nuts and seeds. All processed foods contain food additives to extend shelf-life, increase taste and increase convenience during production [34]. Examples of common processed foods include breakfast cereals, cakes, biscuits, tinned vegetables and meat products such as sausage and corned beef.

Whole foods have high nutrient density values that contain carbohydrates, proteins, fats, vitamins and minerals. Grain foods such as rice and highly starchy vegetables such as potatoes are a source of carbohydrates [35]. The World Health Organization recommend that people’s daily carbohydrate intake should be around 55 to 75% of their total energy [36].

Animal-based foods such as poultry, pork and beef contain all the essential amino acids that the body needs. In addition, eating a wide variety of plant-based foods such as nuts and seeds can also provide essential amino acids for the body [35]. It is recommended that daily protein intake should be around 10 to 15% of total energy [36]. Meat contains saturated fat, and nuts and seeds contain unsaturated fat [35]. It is recommended that fat intake should be limited to 15 to 30% of total daily energy, of which saturated fat should not exceed 10% and trans fat should not exceed 1% [36].

Fruits and vegetables are rich in dietary fibre, vitamins and minerals such as vitamins B, C and potassium [35]. Adolescents and adults should consume 25 grams or more of dietary fibre per day in daily life [36].

Most processed foods are energy-dense and contain high calories, high fat, high sugar and sodium. In a daily diet, it is not suggested to consume more than 5 grams of sodium, and daily sugar intake should not exceed 50 grams [36]. Some processed foods, such as cakes and sodas, have added sugar called empty calories that do not contain any nutrients [33]. Therefore, people should eat less canned or preserved foods, such as pickles and sausages. Moreover, natural condiments, such as ginger and pepper, should be chosen during cooking to reduce sugar and salt intake in daily living.

### 4.2. Health Impacts of Dietary Habits

A balanced diet is beneficial and it is the key to good health. On the contrary, an unbalanced diet will increase the risk of chronic diseases. Diet-related chronic diseases include hypertension, diabetes and CVD. The Centre for Health Protection (2015) has found that one tenth of people aged 15 years or above eat at least one processed food every day. In addition, most people eat less than five portions of fruits and vegetables every day [37]. It appears that people are now consuming food that is high in energy, fat and sodium and low in fruits and vegetables.

Sodium intake is directly related to blood pressure [38]. High sodium intake leads to rising blood pressure and the risk of hypertension [36]. Compared to unprocessed meats, processed meats contain more sodium [39]. Limiting sodium intake and increasing the consumption of fruits, vegetables and low-fat dairy products can reduce the risk of hypertension. Several studies have found that limited sodium intake can reduce blood pressure in people with or without hypertension [38]. With the reduction of hypertension, the risk of CVD is reduced by changing blood pressure distribution [36].

Meat is a source of saturated fat and saturated fatty acids [39]. Epidemiological studies have found that a high consumption of saturated fat and saturated fatty acid foods, such as processed meats, decrease glucose tolerance and insulin sensitivity and increase fasting glucose [36], but the consumption of unprocessed meat is not associated with the risk of diabetes [39]. In addition, some controlled experimental studies have also found that the consumption of unsaturated fatty acids, such as vegetables, can improve glucose tolerance and increase insulin sensitivity. Dietary fibre can reduce blood glucose and insulin levels in people with diabetes by consuming grain foods, vegetables and fruits [36]. A cross-sectional study conducted by Ibarrola-Juardo et al., which involved 1068 older adults with a mean age of 67 (±6) as the study subjects, reported that dietary intake of phylloquinone in leafy green vegetables was associated with a lower prevalence of type II diabetes. Increased phylloquinone intake in the follow-up, with a median of 5.5 years, was associated with a 51% reduction in the risk of diabetes onset in the elderly at high cardiovascular risk [24].

High low-density lipoprotein (LDL) cholesterol and low high-density lipoprotein (HDL) cholesterol levels increased the risk of CVD such as coronary heart disease (CHD) and stroke [39]. Several cohort studies have stated that the consumption of trans fatty acids such as deep-fried fast foods and baked goods can increase LDL cholesterol and lower HDL cholesterol levels, thus increasing the risk of CVD [36]. Dietary fibre can reduce LDL cholesterol and nuts are high in unsaturated fatty acids and low in saturated fats; therefore, the frequent consumption of dietary fibre and nuts can reduce the risk of CHD. In addition, fish consumption can reduce mortality and morbidity rates of the disease [36].

All in all, whole foods can reduce the risk of hypertension, diabetes, and CVD. It is important to build healthy diet habits to maintain health. Consuming more natural foods such as fruits, vegetables, grain foods and low-fat dairy products instead of processed foods is good for health.

### 4.3. Food and Health among the Elderly

As nutrition is a critical factor affecting health and well-being, adopting a balanced diet with appropriate planning is essential for healthy ageing, considering the association between dietary patterns and chronic conditions due to ageing [40,41]. Since the late 1900s, there have been numerous studies on dietary patterns, such as the Eskimo diet and Japanese diet. It has been found that a high intake of fish can reduce the risk of CHD due to it being rich in n-3 and n-6 PUFA [42]. Various reports have indicated the important health and/or environmental implications of a balanced diet emphasising plant-based foods [10,43,44]. Substantial evidence from epidemiological studies supports the association of consuming diets abundant in plant foods with a reduction in the risk of CVD, which is a prevailing chronic illness for the elderly [45,46]. Recent reviews also reported that diets rich in fruits and vegetables were associated with a decreased risk of CHD and cancers, leading to generalised recommendations on public health to increase intakes of fruits and vegetables [9,47,48].

Furthermore, the extensive review by Lutz et al. (2019) described the crucial health benefits of phenolics, which are specific secondary metabolites in plants and are reported to have various beneficial health effects [49]. Over the past decades, the potential health benefits of phenolic compounds have been one of the hot topics in the research of human health [50]. The protective roles of dietary phenolics in the prevention of chronic diseases such as CVD and certain cancers have been extensively studied. The findings support the beneficial health effects of dietary phenolics including antiplatelet activity [51], antioxidative properties [52] and anti-inflammatory activity [53,54,55]. Furthermore, plant-based diets which provide a reliable source of phenolic compounds are reported to have a potential role in lowering the risk of cognitive disorders [56,57,58].

Although plant-based foods have chemical constituents which can bring beneficial effects to human health, there are other nutrient sources required by the elderly for healthy ageing. Functional impairment and disability are common in the elderly due to a gradual weakening of muscle strength and loss of muscle mass [59], leading to limited capabilities of performing daily-life tasks, including self-care [60]. Therefore, daily protein intake is essential for the elderly to facilitate the synthesis of muscle protein and enhance mitochondrial function [61], which has an important role in keeping lean body mass [62]. Although there are various dietary sources of protein such as vegetable and animal proteins, those from animal sources, for example, meat and poultry, are considered high quality proteins [63]. However, meat consumption, with both good and bad nutritional attributes, has been a highly controversial subject in research agenda and public debate [64]. On one side, meat is an important micronutrient-rich food and a significant dietary source of high-quality protein. On the other, high consumption of meat has been widely blamed for the prevalence of chronic diseases, with specific concern over the category of red meat and processed meat [65]. For example, colorectal cancer, one of the leading diagnosed malignancies in different countries over the world, has been linked with the consumption of red and processed meat [66]. Meanwhile, dietary meat fats are reported to have also contributed to the burden of CVD among the elderly [67,68]. Overall, there are substantial findings which support the association of meat consumption, especially for red meat and processed meat, with the increased risk of chronic diseases.

In 2013, the WHO recommended a reduction of the population’s salt intake with a global target of 30%, which aimed to ultimately achieve an overall 25% reduction of premature mortality due to non-communicable diseases by the year of 2025 [69]. Since then, there has been an increasing number of salt reduction programmes and initiatives with implementation of different structural or regulatory approaches throughout the world. Comprehensive reviews have elegantly summarised the recent progress and effectiveness of nationwide salt reduction initiatives or strategies by different countries [11,12,70,71,72]. It is noteworthy that state-level and community-level efforts on the reduction of salt intake are also crucial with related interventions demonstrating a certain degree of effectiveness [73]. Overall, interventions for the reduction of salt intake are of particular significance to the elderly since they are at a higher risk of hypertension. Industry-level interventions alone are not enough. The reduction of salt intake should also take place through individual interventions for effective control. After all, individual awareness is important for a person to opt for food with reduced sodium levels. However, certain obstacles exist due to various factors.

For elderly people, they have a lower sensitivity in the detection of taste in general when compared to younger adults [74]. A reduction in salt sensitivity is also observed for people experiencing hypertension [75]. The loss of salt sensitivity during ageing can lead to the wrong perception and judgment of saltiness, and subsequently higher levels of salt intake by the elderly [76]. In fact, people can add salt by themselves when dining at the table, as compensation for the perception of reduced saltiness of food [16,77]. Furthermore, the situation is complicated by the reluctance of people to change their dietary behaviours even though they know such behaviours are not good for health. One of the reasons is the preference of people towards taste rather than health concerns [78]. In addition, it has been reported that some older males had a reluctance to lower salt intake, even when they were presented with the benefits of reducing salt consumption [16,79].

Added to that, environmental concerns also arise in terms of climate change associated with vast meat consumption. Meat production is a contributor of greenhouse gas emissions and climate change [64,80]. Put forth as part of the strategies to mitigate climate change, recommendations have been made on dietary habits which include the reduction of meat consumption, while the importance of controlling meat consumption has also been discussed in recent reports [64,80]. Findings also indicate that consuming less meat while meeting the standard requirement for health can lower the emission of greenhouse gases [81,82]. Considering the environmental and health concerns, sustainable diets based on plant foods should be advocated for more in the pursuit of better health and sustainable environment to promote healthy ageing.

### 4.4. Green Diet and Healthy Ageing

In recent years, the green diet has received growing attention. A green diet means the consumption of a diet rich in plant foods with minimal portions of meat, and at the same time emphasising the importance of food origins to be environmentally responsible [17]. The word “green” also implies consumption of food in an appropriate amount to prevent food wastage. A clear mindset to reduce food waste is imperative, and with careful planning excessive and unnecessary purchases of food can be avoided. With reduced meat consumption being an important feature of the green diet, it should be noted that the promotion of the green diet is not an easy task. In the study by Macdiarmid et al. [64], it was revealed that some people held the belief that individual reduction of meat consumption would have a very slight contribution to help mitigate climate change. It was also found that the participants in general were not aware of the relationship between the extent of meat consumption and change of climate. General resistance to reduced meat consumption in a diet still existed even after they had been presented with the consequences. In fact, consuming meat in a diet is a traditional and major eating pattern, and has been long considered as a biocultural activity in many places [83,84]. The reasons for eating meat can be psychological, giving the sensation of pleasure or symbolic implications of social or economic status [85]. It is important that the cultural and social issues surrounding meat consumption are not overlooked, as they have considerable influence on a person’s readiness to alter their individual dietary habits. Considering personal, social and cultural values, these explain the probable reasons behind the polarised general public views of meat consumption, even when there are substantial findings supporting the associations of red and processed meat consumption with non-communicable diseases [44], such as CVD [86], colorectal cancer [87] and type II diabetes [86,88].

The practice of a green diet should also emphasise healthy dietary habits, which are crucial for the promotion of healthy ageing. For example, a reduction of salt intake helps to control hypertension [89], which is a common chronic disease in the elderly and is also closely related to CHD [90]. Numerous findings have reported that excessive intake of salt leads to high blood pressure [91,92], and interventions to control population salt intake are necessary for reducing the risk factors of associated chronic diseases [93]. Meanwhile, there are reports which show that reducing a population’s salt intake is one of the most cost-effective interventions to relieve the burden of non-communicable diseases [12,94].

In short, following a green diet with good dietary practices is important to human health, especially for elderly people who are at a high risk of various chronic diseases such as CHD, diabetes and certain cancers. As discussed in previous paragraphs, a diet rich in plants with low portions of meat is associated with beneficial health effects against chronic conditions. Enriching a diet with more green plant foods and a lower red meat portion is beneficial to health, which was also reported by a recent study investigating the positive effects on cardiometabolic risk by following a green Mediterranean diet [95]. While a green diet symbolises a dietary habit of adopting healthy dietary practices which addresses the importance of a green environment, both the green diet and the traditional Mediterranean diet emphasise high consumption of leafy green vegetables, with the latter placing particular importance on the consumption of olive oil [96,97]. Recently, Álvarez-Álvarez et al. reported the results of a cross-sectional study involving 6874 older adults which showed that a higher adherence to a Mediterranean diet was associated with a reduced prevalence of cardiovascular risk factors [22]. Meanwhile in 2019, Jennings et al. also reported that an adherence to a Mediterranean diet resulted in improved cardiovascular health with reduced blood pressure and arterial stiffness [25]. Furthermore, the study by Chan et al. in 2019, which encompassed a community cohort of 2802 elderly participants with a mean age of 73, revealed that a higher Diet Quality Index-International (DQI-I) score and an Okinawan diet score were associated with a reduction in the risk of all-cause mortality, while a higher Mediterranean-diet intervention for neurodegenerative delay (MIND) score was linked with a lower risk of CVD mortality [29]. Numerous independent studies in Italy, Spain, United Kingdom, Japan and the United States of America also reported that a Mediterranean diet (rich in plants, fish and olive oil but low-fat dairy products) was associated with a lower risk of CHD [98,99,100,101,102]. Another comprehensive review from Salehi-Abargouei et al. (2013) on the dietary approaches to stop hypertension (DASH) diet (rich in fruits, vegetables, low-fat dairy products, grains, poultry, fish and nuts, and low in saturated fat, red meat, sweets and sugar-containing beverages) also reported that the risk of CVD, CHD, stroke and heart failure among middle-aged and older people was reduced [103].

While there are established findings supporting the association of plant-based diets with a reduction in the risk of chronic diseases, recent studies also supported the link with delayed cognitive function decline among the elderly, which leads to a general improvement in quality of life [104]. Similar findings were also reported on the Mediterranean diet [23,26,27,30,31,105,106]. In addition, higher adherence to a MIND diet was associated with a lower prevalence of parkinsonism. The study by Agarwal et al. in 2018 reported that a unit increase in a MIND diet score was associated with a reduction of 13% for the rate of parkinsonism development [28]. Furthermore, the study by Morris et al. (2015) on the Mediterranean-Dietary Approach to Systolic Hypertension (DASH) diet intervention for neurodegenerative delay (MIND) reported that the MIND diet slowed down cognitive decline with ageing. This would also bring considerable benefits to caregivers when there is a delay of decline in cognitive functions of the elderly [107]. It is also critical to healthy ageing, considering the prevalence of cognitive decline and dementia among the ageing population. Meanwhile, it should be noted that the green diet is not advocating a meal with a zero portion of meat. Rather, the practice of a minimised amount of red meat is emphasised.

Going green on a diet not only results in profound benefits to human health, it also contributes to a sustainable environment, which is also important for healthy ageing. However, promoting a green diet is not just about disseminating information to the public with relevant benefits and new findings. For the promotion of healthy ageing, the psychological well-being of the elderly should also be taken into account. After all, the feelings of a person in choosing a dietary pattern should not be overlooked. The change of dietary habits is not easy, especially for an elderly person who might have been adopting certain dietary patterns for many years. Similar to the practice of reduction in salt intake, even with the established findings of substantial health benefits, consumption of a low-meat diet may not be favoured due to individual taste concerns and persistent dietary preference. Therefore, community health promotion including education programmes at elderly care centres is expected to play an important role.

### 4.5. Recommendations and Implications for Research Studies

More investigations are required to make further advancement in the knowledge and analysis of the active chemical components affecting human health from fruits and vegetables. Dietary patterns play an important role in the promotion of healthy ageing. More scientific evidence of the beneficial health effects of phenolic compounds by clinical studies would help promote the association of phenolic compounds with healthy ageing. For example, the effects on the activities of phenolic compounds by thermal processing of foods is worth further investigations [49]. In addition, more dietary guidelines should be developed to incorporate the concepts of a green diet and sustainable environment. Both health and environmental concerns are important and should be considered when planning appropriate dietary patterns. While more research in behaviour change theories and sustainable diets are encouraged, collaborative efforts by the food industry, health professionals and governments are also imperative and they should be responsible for concurrent actions to provide consumers with healthy and sustainable choices of diets in terms of accessibility and equity [108]. Meanwhile, it has been reported that there is a significant association of dietary knowledge with the dietary behaviours and attitudes of the elderly [109]. A higher level of nutritional knowledge is linked with the appropriate intake of vegetables and fruits. Therefore, community health promotion and education are important in public health initiatives to increase self-awareness and health literacy, and equip the elderly with sufficient dietary knowledge to choose healthy eating habits and go for a green diet.

The adoption of a green diet includes the practice of good dietary habits. Specific programmes and initiatives, at both the national and community levels, are expected for the promotion of healthy ageing. Reducing meat consumption should also be identified as a significant personal change one can make for the alleviation of climate change. Meanwhile, a recent study researching the effects of messages on reducing meat consumption by elderly people revealed that the content and the framing of persuasive messages should be considered together for the better planning and design of communication campaigns [110]. More research studies are necessary for examining the conditions and parameters under which effective communication regarding nutrition with the elderly can be achieved, thus leading to the promotion of changes in some unhealthy eating habits of the elderly.

Hypertension is highly prevalent among the elderly and salt reduction interventions are of prominent significance to reduce the burdens from associated chronic diseases. As discussed in previous sections, there exists barriers in the reduction of a population’s salt intake. Technological advances are required for maintaining the taste of food with reduced sodium levels [16]. Furthermore, considering the worldwide salt reduction initiatives, close monitoring and more evaluations, as well as the sharing of experience in relevant implementations by different countries, are expected in the near future.

## 5. Conclusions

In conclusion, diet and nutrition are critical factors affecting human health and well-being. With the worldwide growth of the ageing population, the promotion of good dietary patterns is particularly important among the elderly with the objective of relieving the burden of chronic diseases. A green diet can bring beneficial effects to both human health and the environment as an initiative of global sustainable development [111], and is expected to give significant contributions to healthy ageing. Overall, more research and interventions are required for the promotion of good dietary practices including the consumption of minimised meat portions in a diet and the reduction of daily salt intake.

## Figures and Tables

**Table 1:** Summary table of studies investigating the dietary pattern and health effects among the elderly.

Reference Number	Author and Year	Country/Region	Intervention Type	Sample Size	Age	Relevant Findings
[22]	Álvarez-Álvarez et al., 2019	Spain	Cross-sectional	6874	Men: 55 to 75; women: 60 to 75	Higher adherence to a Mediterranean diet was associated with a reduced prevalence of cardiovascular risk factors.
[23]	Hardman et al., 2015	Australia	Randomised controlled trial	148	60 to 90	Mediterranean diet interventions and exercise, alone or combined, improved cognitive performance.
[24]	Ibarrola-Jurado et al., 2012	Spain	Cross-sectional	1068	Mean age: 67 ± 6	Dietary intake of phylloquinone in leafy green vegetables was associated with a lower prevalence of type II diabetes. Increased phylloquinone intake in the follow-up, with a median of 5.5 years, was associated with a 51% reduction in the risk of diabetes onset in the elderly at high cardiovascular risk.
[25]	Jennings et al., 2019	Participants were recruited from centres in 5 countries (Italy, United Kingdom, Netherlands, Poland and France)	Clinical trial	1294	65 to 79	Adherence to a Mediterranean diet resulted in improved cardiovascular health with reduced blood pressure and arterial stiffness.
[26]	Knight et al., 2015	Australia	Randomised controlled trial	166	65 or above	The positive association between adherence to a Mediterranean diet and reduced cognitive decline was validated.
[27]	Tussing-Humphreys et al., 2017	United States	Randomised controlled trial	180	55 to 85	A Mediterranean dietary pattern was associated with a reduction in the risk of cognitive impairment and dementia.
[28]	Agarwal et al., 2018	United States	Longitudinal cohort study	706	59 to 97	Higher adherence to a MIND diet was associated with a lower prevalence of parkinsonism. A unit increase in a MIND diet score was associated with a reduction of 13% for the rate of parkinsonism development.
[29]	Chan et al., 2019	Hong Kong	Community cohort study	2802	Mean age: 73	A Higher Diet Quality Index-International (DQI-I) score and Okinawan diet score were associated with a reduction in the risk of all-cause mortality, while a higher MIND score was linked with a lower risk of CVD mortality.
[30]	Limongi et al., 2017	Italy	Longitudinal study	5632	65 to 84	High adherence to a Mediterranean diet was associated with a reduced risk of all-cause mortality and a lower prevalence of emotional impairment and cognitive decline.
[31]	Trichopoulou et al., 2015	Greek	Longitudinal study	816	65 or above	Evidence supported the protective effect of higher adherence to a Mediterranean diet against cognitive impairment over 7 years of extended period, especially in the elderly aged 75 years or older.
